# Molecular characterization of carbapenem and ceftazidime-avibactam-resistant Enterobacterales and horizontal spread of *bla*_NDM-5_ gene at a Lebanese medical center

**DOI:** 10.3389/fcimb.2024.1407246

**Published:** 2024-06-19

**Authors:** Ghena Sobh, George F. Araj, Marc Finianos, Tsolaire Sourenian, Jaroslav Hrabak, Costas C. Papagiannitsis, Mira El Chaar, Ibrahim Bitar

**Affiliations:** ^1^ Department of Pathology and Laboratory Medicine, American University of Beirut Medical Center, Beirut, Lebanon; ^2^ Department of Microbiology, Faculty of Medicine, University Hospital in Pilsen, Charles University, Pilsen, Czechia; ^3^ Department of Microbiology, University Hospital of Larissa, Larissa, Greece; ^4^ Faculty of Health Sciences, University of Balamand, Beirut, Lebanon

**Keywords:** *Escherichia coli*, *Klebsiella pneumoniae*, ST383, bla_NDM-5_, carbapenem resistance

## Abstract

**Introduction:**

In the battle against multidrug-resistant bacterial infections, ceftazidime- avibactam (CZA) stands as a pivotal defense, particularly against carbapenemresistant (CR) Gram-negative pathogens. However, the rise in resistance against this drug poses a significant threat to its effectiveness, highlighting the critical need for in-depth studies about its resistance mechanisms.

**Methods:**

This research focuses on the genomic characterization of CR- and CZA-resistant *Escherichia coli* (n=26) and *Klebsiella pneumoniae* (n=34) strains, harboring the blaNDM and/or blaOXA-48-like genes, at a major Lebanese tertiary care medical center, using whole genome sequencing (WGS).

**Results:**

Our findings revealed a notable prevalence of blaNDM in all *K. pneumoniae* strains isolates, with 27 of these also harboring blaOXA-48. On the other hand, E. coli strains predominantly carried the blaNDM-5 gene. Whole genome sequencing (WGS) identified a predominance of ST383 among K. pneumoniae strains, which possessed a multi-replicon IncFIB-IncHI1B plasmid harboring the blaNDM-5. Additionally, various Inc group plasmids in *K. pneumoniae* across multiple sequence types were found to carry the blaNDM. Similarly, diverse STs of E. coli were observed to carry blaNDM-5 on different plasmids.

**Discussion:**

The study underscores NDM carbapenemases as a paramount resistance mechanism in Lebanon,jeopardizing critical last-resort treatments. It also illuminates the role of varied sequence types and mobile genetic elements in the spread of NDM resistance,stressing the urgent need for strategies to mitigate this threat, especially in nosocomial infections.

## Introduction

Carbapenems are the drug of choice for the treatment of complicated multidrug-resistant (MDR) bacterial infections. However, their misuse has led to an increased emergence of resistant organisms (Gupta et al., 2011). The World Health Organization (WHO) has classified carbapenemase-producing Enterobacterales (CPE), especially *Klebsiella pneumoniae* and *Escherichia coli*, as a worldwide public health concern due to their proliferation in hospital settings (https://apps.who.int/iris/bitstream/handle/10665/312226/WHO-UHC-SDS-2019.6-eng.pdf?sequence=1&isAllowed=y). Resistance mechanisms to carbapenems include the increased activity of the efflux pumps, porin loss, and β-lactamases production, particularly carbapenemases (Ye et al., 2018). These carbapenemases are divided according to Ambler into four classes, with only class A [i.e., *Klebsiella pneumoniae* carbapenemase (KPC)], Class B [i.e. New-Delhi Metallo-β-lactamase (NDM)], and Class D [i.e., Oxacillinase (OXA)-48 and OXA-48-like] being associated with carbapenemase activity (Ambler, 1980; van Duin and Doi, 2017; Kopotsa et al., 2019).

NDM is among the most common and clinically significant carbapenemase in Enterobacterales capable of efficiently hydrolyzing a wide range of β-lactams, including penicillins, carbapenems, and cephalosporins (Nordmann et al., 2011; Nordmann and Poirel, 2014). It has rapidly spread across the Middle East, the Indian subcontinent, and the Balkan region (Dortet et al., 2014; Wu et al., 2019). In the United Kingdom, in 2011, NDM-5 was first detected, and two amino acid substitutions at positions 88 and 154 lead to increased NDM enzymatic activity (Hornsey et al., 2011).

Furthermore, there has been a notable spread of genes encoding OXA-48-like carbapenemases. In 2001, a *K*. *pneumoniae* strain resistant to all available β-lactams was reported in Turkey. A subsequent study by Poirel et al. in 2004 identified this isolate as carrying a novel *bla* variant, *bla*_OXA-48_ (Poirel et al., 2004). Isolates harboring *bla*_OXA-48-like_ genes have garnered attention due to their rapid horizontal transmission and increasing detection across the Middle East and North Africa showing resistance to β-lactamase inhibitors (Poirel et al., 2004; Nordmann and Poirel, 2014; Al-Zahrani and Alsiri, 2018; Mairi et al., 2018). Co-production of NDM- and OXA-48-like enzymes has been increasingly reported, particularly in patients with travel histories from the Middle East, Southeast Asia, Europe, China, and Africa (Doi et al., 2014; Avolio et al., 2017; van Duin and Doi, 2017; Otlu et al., 2018; Solgi et al., 2020; Chudejova et al., 2021; Lorenzin et al., 2022).

In Lebanon, few studies highlighted the prevalence of carbapenem resistance (CR) and their mechanisms among clinical isolates in this country (Baroud et al., 2013; Araj et al., 2018). For example, a study conducted between 2008 and 2012 showed an increase in reduced susceptibility to ertapenem from 0.4% to 1.6% (Beyrouthy et al., 2014). By 2019, resistance levels in Enterobacterales reached 3.3%, with higher rates observed in *Pseudomonas* spp. (27.3%) and *Acinetobacter* spp. (53.7%) (Rima et al., 2022). In 2008, the first case of OXA-48 was detected in *K*. *pneumoniae* in Lebanon (Matar et al., 2008). Moreover, the first cases of NDM-1 and NDM-5, isolated in 2010 and in 2017, respectively, were identified in *K. pneumoniae* isolates (El-Herte et al., 2012; Nawfal Dagher et al., 2019). Between 2015 and 2019, carbapenemase-producing *E. coli* were found to be positive for *bla*_OXA-48_ (n=11), *bla*_OXA-181_ (n=12), and *bla*_NDM-5_ (n=4) and *bla*_NDM-19_ (n=9), while *K. pneumoniae* isolates harbored *bla*_OXA-48_ (n=6), *bla*_OXA-181_ (n=1), and *bla*_NDM-5_ (n=1) (Bitar et al., 2018; Rima et al., 2022). A recent study in Lebanon in 2019, following the introduction of ceftazidime-avibactam (CZA) as a treatment for extended spectrum β-lactamase (ESBL) and multi-drug resistant (MDR) infections, analyzed 150 isolates (*E. coli* and *K. pneumoniae*). These were classified as ESBL (n=20), MDR (n=20), or carbapenem resistant (CR) (n=110). All CR isolates exhibited a 100% resistance to ertapenem. Moreover, 60% (n=30) of CR *E. coli* and 65% (n=39) of CR *K. pneumoniae* showed resistance to CZA, with all resistant isolates being producers of NDM and/or OXA-48-like enzymes (Sobh et al., 2021).

This study, thus, was warranted at expanding our understanding of the resistance and dissemination by performing a whole genome characterization of CR- and CZA-resistant *E*. *coli* and *K*. *pneumoniae* strains producing NDM and/or OXA-48-like carbapenemases, at a major Lebanese tertiary care medical center.

## Materials and methods

### Bacterial isolates and determination of carbapenems minimal inhibitory concentrations

This study encompassed characterization a total of 60 Enterobacterales isolates (consisted of 34 isolates of *K*. *pneumoniae* and 26 isolates of *E*. *coli*) recovered at the Clinical Microbiology Laboratory of the Department of Pathology and Laboratory Medicine at the American University of Beirut Medical Center, between 2019 and 2021. These isolates as were identified by the matrix-assisted laser desorption ionization-time of flight mass spectrometry (MALDI-TOF MS) using the MALDI-biotyper software (Bruker Daltonics, Bremen, Germany). The MICs determination to three carbapenems and CZA was carried out as reported earlier (Sobh et al., 2021).

For further characterization, all isolates were sent to Charles University, Faculty of Medicine in Pilsen, Czech Republic. Carbapenemase production and activity were assessed using MALDI-TOF MS with the meropenem hydrolysis assay, as described by Rotova et al. (Rotova et al., 2017), and the double disk synergy test incorporating EDTA, phenylboronic acid, and temocillin, following previously described methodologies (Lee et al., 2003). In addition antimicrobial susceptibility testing was conducted using the broth microdilution method, adhering to the EUCAST guidelines, with results interpreted based on the EUCAST 2024 criteria (https://www.eucast.org/clinical_breakpoints) (Clinical breakpoints - breakpoints and guidance v13.0, 2023).

### Gene content and multilocus sequence typing by PCR

The presence of *bla*_IMP_-like, *bla*_KPC_-like, *bla*_NDM_-like, *bla*_VIM_-like, and *bla*_OXA-48_-like genes was tested via PCR (Papagiannitsis et al., 2015). Additionally, multilocus sequence typing (MLST) was performed on the isolate collection as previously described (Diancourt et al., 2005; Wirth et al., 2006; Brisse et al., 2009).

### Short-reads sequencing and analysis

Based on MLST and MIC results, 17 K*. pneumoniae* and 10 *E. coli* isolates were selected as representatives for whole genome sequencing. Genomic DNA was extracted using NucleoSpin Microbial DNA kit (Machery-Nagel, Germany). A multiplexed library was prepared using Nextera XT library preparation kit, and sequencing was performed on the Illumina MiSeq platform (Illumina Inc., San Diego, CA, United States) using the MiSeq v3 reagent kit using 600 cycle cartridges. Initial paired-end reads were quality assessed using fastqc, then trimmed and filtered to high quality using fastp (Andrews, 2010; Chen et al., 2018). Subsequent reads were assembled by a de Bruijn graph-based *de novo* assembler SPAdes (Prjibelski et al., 2020). The assembled contigs were analyzed to detect sequence types (STs) using MLST software, virulence genes using VFanalyzer, resistance genes using ResFinder, and plasmid incompatibility (Inc) types using PlasmidFinder (Carattoli et al., 2014; Liu et al., 2019; Florensa et al., 2022). Additionally, these contigs were also uploaded to PHASTER to detect phage genomes, CRISPRfinder to detect CRISPR-cas systems, and ISfinder to detect insertion sequences (Siguier et al., 2006; Arndt et al., 2016).

### Long-read sequencing and analysis

Based on the previous short-read results, seven and four representative isolates corresponding to *K. pneumoniae* and *E. coli* isolates, respectively, were selected for long-read sequencing using PacBio Sequel I (Pacific Biosciences, CA, United States). Briefly, the extracted DNA was subjected to shearing using the Megaruptor 2 (Diagenode, Liege, Belgium); this will generate sheared fragments of ~15 kb each. Library preparation was performed using the microbial multiplexing protocol according to the manufacturer’s recommendation. During the library preparation, no size selection was performed. Using SMRT Link v10.1, the microbial assembly pipeline was used for the assembly and the circularizing of contigs using a minimum seed coverage of ×30. The assembled contigs were then analyzed using the same procedures as the short-read assembly. Finally, all genomes were annotated using the NCBI’s Prokaryotic Genome Annotation Pipeline (Tatusova et al., 2016; Haft et al., 2018; Li et al., 2021).

### Phylogeny and single nucleotide polymorphism

To study the genetic diversity and phylogenetic relationship of the studied isolates and global isolates, genomes of *K. pneumoniae*, specifically of the ST383 type, were retrieved from the NCBI assembly database, encompassing both complete and draft genomes, totaling 105 genomes. Using parsnp v1.7.4 (available in the harvest suit), a core genome phylogeny of the model General Time Reversible (GTR) based on SNP and recombination, an SNP-based phylogeny was constructed between the sequenced genomes in this study and the 105 genomes downloaded from the NCBI database using KP1674 as a reference (complete circular genome) and *K. quasipneumoniae* as an outgroup (Edgar, 2004; Bruen et al., 2006; Price et al., 2010; Treangen et al., 2014). Briefly, SNPs identified in local collinear blocks were subsequently used for reconstructing an approximate maximum-likelihood tree using FastTree, while including the general time reversible (GTR) model of nucleotide substitution. The Shimodaira–Hasegawa test implemented in FastTree2 was used to assess the support for significant clustering in the observed phylogeny. The interactive tree of life or iTOL (https://itol.embl.de/) was used for the graphic illustration of the trees along with relative annotations.

Moreover, the SNPs between the ST383 genomes were detected to determine the extent of clonal dissemination in the hospital. SNPs were detected using snippy multicommand (snippy-base application v4.5.0) (Seeman, 2015), which generates a core genome multiple alignment against the common reference (KP1674). The pipeline detects the variants and generates a single file for each isolate listing the different variations.

### Comparison of antimicrobial multidrug-resistant region

The MDR sequence from KP1633 isolate (complete circular genome) was used as a reference to compare with the rest of the isolates. Easyfig v2.2.0 was used for generating the figure (Sullivan et al., 2011). It uses blastn from the package blast + by NCBI to generate a similarity report between the fragments (Camacho et al., 2009). An alignment between the short-read sequences and the MDR sequence characterized with Pacbio was used to generate a consensus and then annotated using Prokka to include in the generation of the figure (Seemann, 2014).

### Data availability

All genomes have been submitted to the NCBI database. Accession numbers are available under the BioProject PRJNA952851.

## Results

### Phenotypic antimicrobial resistance profile of the isolates

All 34 K*. pneumoniae* isolates showed resistance against CZA, carbapenems, tazobactam, ciprofloxacin, and tetracycline. Moreover, all isolates showed resistance against amikacin and gentamicin (except one isolate) (**Table 1**). Similarly, all *E. coli* isolates (n=26) were also resistant against CZA, carbapenem, and showed resistance to ciprofloxacin and tazobactam and tetracycline (except two isolates). However, all isolates were susceptible against amikacin apart from two resistant isolates (**Table 2**).

**Table 1:** MIC and disk diffusion test results for the *K. pneumoniae* isolates.

Isolate ID				MIC (µg/ml)				DD (S, I, R)								
	Date	Specimen	Ward	Ert	Imp	Mero	CZA	Amk	Cip	Gent	Tazo	Tet	Trimeth	Tige	Col	Fosfo
KP1601	10/01/2020	Urine	ICU	>32	>32	>32	>256	R	R	R	R	R	R	–	I	R
KP1608	27/01/2020	Urine	OC	>32	4	16	>256	R	R	R	R	R	R	–	I	R
KP1612	01/02/2020	Blood	ICU	>32	>32	>32	>256	R	R	R	R	R	R	S	I	I
KP1618	10/02/2020	Urine	8 north	>32	>32	>32	>256	R	R	R	R	R	R	–	I	R
KP1623	17/02/2020	DTA	ICU	>32	>32	>32	>256	R	R	R	R	R	R	S	I	R
KP1633	01/03/2020	DTA	5 south	>32	>32	>32	>256	R	R	R	R	R	R	S	I	I
KP1635	06/03/2020	DTA	ICU	>32	>32	>32	>256	R	R	R	R	R	R	S	R	R
KP1639	09/03/2020	Fluid	5 south	>32	>32	>32	>256	R	R	R	R	R	R	S	I	I
KP1642	12/03/2020	Blood	9 north	>32	>32	>32	>256	R	R	R	R	R	R	–	I	R
KP1645	20/03/2020	Urine	ICU	>32	>32	>32	>256	R	R	R	R	R	R	–	R	R
KP1648	10/04/2020	Urine	ICU	>32	>32	>32	>256	R	R	R	R	R	R	–	I	R
KP1652	20/04/2020	Blood	ICU	>32	>32	>32	>256	S	R	R	R	R	R	S	I	R
KP1655	21/04/2020	Tissue	ICU	>32	>32	>32	>256	R	R	R	R	R	S	R	R	–
KP1656	23/04/2020	Urine	ICU	>32	>32	>32	>256	R	R	R	R	R	R	–	I	S
KP1657	24/04/2020	Blood	NEUROICU	>32	>32	>32	>256	R	R	R	R	R	R	–	–	–
KP1658	30/04/2020	DTA	ICU	>32	>32	>32	>256	R	R	R	R	R	R	S	I	I
KP1661	05/05/2020	Wound	5 south	>32	>32	>32	>256	R	R	R	R	R	R	S	–	–
KP1663	17/05/2020	Urine	9 south	>32	>32	>32	>256	R	R	R	R	R	R	–	I	S
KP1669	23/05/2020	DTA	ICU	>32	>32	>32	>256	R	R	R	R	R	R	S	I	I
KP1674	25/05/2020	DTA	10 south	>32	>32	>32	>256	R	R	R	R	R	S	–	I	I
KP1679	01/06/2020	Sputum	9 south	>32	>32	>32	>256	R	R	R	R	R	R	S	I	S
KP1688	17/06/2020	Urine	BASIL IN	>32	>32	>32	>256	R	R	R	R	R	R	–	R	I
KP1690	28/06/2020	Screening	NEUROICU	>32	>32	>32	>256	R	R	R	R	R	R	S	S	R
KP1696	02/07/2020	Urine	9 north	>32	>32	>32	>256	R	R	R	R	R	R	–	I	–
KP1704	22/07/2020	Blood	BASIL IN	>32	>32	>32	>256	R	R	R	R	R	R	–	S	I
KP1710	06/08/2020	Urine	9 north	>32	>32	>32	>256	R	R	R	R	R	R	–	R	I
KP1734	31/08/2020	Blood	NEUROICU	>32	>32	>32	>256	R	R	R	R	R	R	–	I	R
KP1736	05/09/2020	Sputum	10 south	>32	>32	>32	>256	R	R	R	R	R	R	I	S	S
KP1851	26/02/2021	Screening	6 north	>32	>32	>32	>256	R	R	R	R	R	R	S	I	R
KP1859	08/03/2021	Blood	CCU	>32	>32	>32	>256	S	R	S	R	R	R	R	I	S
KP1877	08/04/2021	DTA	9 north	>32	>32	>32	>256	R	R	R	R	R	R	S	I	S
KP1878	08/04/2021	Screening	NICU	>32	>32	>32	>256	R	I	R	R	R	R	S	I	S
KP1880	08/04/2021	Screening	NICU	>32	>32	>32	>256	R	R	R	R	R	R	S	I	S
KP1917	17/06/2021	Wound	9 south	>32	>32	>32	>256	R	R	R	R	R	R	R	I	R

Ert, ertapenem; Imp, imipenem; Mero, meropenem; CZA, ceftazidim-avibactam; Amk, amikacin; Cip, ciprofloxacin; Gent, gentamicin; Tazo, tazobactam; Trimeth, trimethoprim; Tige, tigecycline; Col, colistin; Fosfo, fosfomycin; CZA MIC, ceftazidim-avibactam minimum inhibitory concentration; DD, disk diffusion; MIC, minimum inhibitory concentration; S, sensitive; I, intermediate; R, resistant; DTA, deep tracheal aspirate; ICU, Intensive Care Unit; OC, outside patient; NEUROICU, Neurology Intensive Care Unit; NICU, Neonatal Intensive Care Unit.

**Table 2:** MIC and disk diffusion test results for the *E. coli* isolates.

Isolates ID				MIC (µg/ml)				DD (S, I, R)								
	Date	Specimen	Ward	Ert	Imp	Mero	CZA	Amk	Cip	Gent	Tazo	Tet	Trimeth	Tige	Col	Fosfo
EC1507	04/10/2019	Tissue Culture	9 north	>32	>32	>32	>256	S	R	S	R	R	R	I	I	S
EC1609	30/01/2020	Urine	6 south	>32	>32	>32	>256	S	R	S	R	R	R	–	S	S
EC1637	07/03/2020	Urine	9 north	>32	>32	>32	>256	S	R	R	R	R	R	–	I	S
EC1649	08/04/2020	DTA	RCU	>32	>32	>32	>256	S	R	S	R	R	R	R	I	S
EC1675	29/05/2020	Urine	10 south	4	1.5	2	>256	S	R	S	R	R	R	–	I	S
EC1723	21/08/2020	Sputum	COVIDICU	32	>32	16	>256	S	R	S	R	S	R	S	I	S
EC1726	22/08/2020	DTA	ICU	>32	>32	>32	>256	S	R	S	R	R	R	S	R	S
EC1733	29/08/2020	Screening	CCU	>32	>32	>32	>256	S	R	R	R	R	R	S	I	S
EC1735	03/09/2020	Urine	8 north	>32	>32	>32	>256	S	R	S	R	R	R	–	S	S
EC1739	11/09/2020	Blood	Basil In	>32	>32	>32	>32	S	R	S	R	R	R	–	I	S
EC1811	12/11/2020	Urine	Basil In	>32	>32	>32	>256	S	R	R	R	R	R	–	I	S
EC1813	17/11/2020	Urine	ICU	>32	>32	>32	>256	S	R	S	R	R	R	–	R	S
EC1825	18/12/2020	Urine	CCU	>32	>32	>32	>256	S	R	S	R	R	R	–	I	S
EC1844	14/02/2021	Blood	Basil In	>32	>32	>32	>256	S	R	S	R	R	R	–	I	S
EC1856	05/03/2021	Urine	7 south	>32	>32	>32	>256	S	R	S	R	R	R	–	I	S
EC1886	17/04/2021	Urine	9 south	>32	>32	>32	>256	S	R	S	R	R	R	–	I	S
EC1911	07/06/2021	Urine	5 south	>32	>32	>32	>256	R	R	R	R	R	R	–	I	–
EC1918	26/06/2021	Blood	9 north	>32	>32	>32	>256	S	R	S	R	R	S	–	R	S
EC1926	15/07/2021	Screening	Basil in	>32	>32	>32	>256	S	R	S	R	R	S	S	S	S
EC1930	31/07/2021	Blood	Basil in	>32	>32	>32	>256	S	R	R	R	R	R	–	R	S
EC1934	03/08/2021	Fluids	Basil in	>32	>32	>32	>256	R	R	R	R	R	R	S	I	S
EC1961	05/09/2021	Urine	COVIDICU	>32	>32	>32	>256	S	R	S	R	R	R	–	I	S
EC1964	14/09/2021	Blood	Basil In	>32	>32	>32	>256	S	R	S	R	R	R	–	S	S
EC1976	29/09/2021	Urine	9 south	8	>32	>32	>256	S	R	S	R	S	R	S	I	S
EC1979	01/09/2021	Blood	9 south	>32	>32	>32	>256	S	R	S	R	R	R	–	I	S
EC1984	12/10/2021	Blood	PICU	>32	>32	>32	>256	S	R	S	R	R	R	–	I	S

Ert, ertapenem; Imp, imipenem; Mero, meropenem; CZA, ceftazidim-avibactam; Amk, amikacin; Cip, ciprofloxacin; Gent, gentamicin; Tazo, tazobactam; Trimeth, trimethoprim; Tige, tigecycline; Col, colistin; Fosfo, fosfomycin; CZA MIC, ceftazidim-avibactam minimum inhibitory concentration; DD, disk diffusion; MIC, minimum inhibitory concentration; S, sensitive; I, intermediate; R, resistant; DTA, deep tracheal aspirate; ICU, Intensive Care Unit; NEUROICU, Neurology Intensive Care Unit; PICU, Pediatric Intensive Care Unit; COVIDICU, COVID-19 Intensive Care Unit; CCU, Critical Care Unit; RCU, Restorative Care Unit; Basil in, Cancer hospital patient.

### Molecular characteristics of the isolates

PCR screening showed that all the *K. pneumoniae* isolates were positive for *bla*_NDM_ (n=34), while 27 of them were simultaneously positive for the presence of *bla*_OXA-48_. On the other hand, the *E. coli* isolates carried *bla*_NDM-5_ gene.

MLST was performed on all the collection. MLST of the *K. pneumoniae* isolates revealed the presence of a major ST: ST383 (n=26), while few other STs were present as well: ST101 (n=2), ST111 (n=1), ST147 (n=1), ST307 (n=1), ST147(n=1), ST39 (n=1), and ST15 (n=1) (**Table 1**). Conversely, based on Achtman Scheme, MLST of the *E. coli* isolates showed several STs circulating within the hospital: ST405 (n=9), ST167 (n=4), ST617 (n=3), ST361 (n=2), ST46 (n=2), ST648 (n=1), ST2450 (n=2), ST131 (n=1), ST998 (n=1), and ST1284 (n=1) (**Table 2**).

### Whole genome sequencing

Based on the susceptibility profiles, antibiotic resistance genes (PCR results), and MLST results, representative isolates (17 K*. pneumoniae* and 10 *E. coli*) were selected for sequencing using short-read sequencing. WGS data showed that 13 of the 17 analyzed *K. pneumoniae* isolates carried *bla*_NDM-5_ while four isolates carried *bla*_NDM-1_. ST383 isolates co-harbored the *bla*_NDM-5_ and *bla*_OXA-48_ genes, while another ST147 isolate co-harbored *bla*_NDM-1_ and *bla*_OXA-232_. All the isolates harbored additional resistance genes against aminoglycoside [*armA*, and/or *aph(3’)-VI*, and/or *aac(6′)-Ib*], beta-lactams (*bla*_SHV-145_, *bla*_OXA-48_, *bla*_NDM-5/1_, and/or *bla*_TEM-1B_), quinolones (*OqxB*, *OqxA*, and/or *qnrS1*), streptogramin b [*msr(E)*], macrolides [*mph(A), mph(E)*], and tetracyclines [*tet(A)*] (**Table 3**). The genomic antibiotic resistance determinants were correlated with the resistant phenotype (**Table 1**). In the 10 analyzed isolates of *E. coli*, WGS data analysis revealed the presence of *bla*_NDM-5_ in all isolates. Moreover, additional resistance genes were detected conferring resistance to beta-lactams (*bla*_NDM-5_, and/or *bla*_CTX-M-15_, and/or *bla*_CMY-145_), macrolides (*mph(A)*), quinolones (the presence of efflux pump AcrAB), aminoglycoside in some isolates [*aac(6′)-Ib-cr*], and tetracyclines [*tet(B)*] (**Table 4** and **Supplementary Table S2**). The genetic antibiotic resistance determinants were associated with the resistant phenotype (**Table 2**).

**Table 3:** WGS data representing resistance gene content and plasmid replicons of the *K. pneumoniae* isolates.

Isolate ID	Sequence Type	*bla*_NDM_ plasmid	*bla*_OXA_ plasmid	Resistance Genes	Other Replicons
KP1658	383	IncFIB–IncHI1B	IncL	*sul1, dfrA5, OqxA, OqxB*, *bla*_OXA-48_, *bla*_CTX-M-15_, *aph(3’)-Ia*, *bla*_SHV-145_, *fosA, aph(6)-Id, aph(3’’)-Ib*, *bla*_CTX-M-14b_, *qnrS1, aph(3’)-VI, mph(E), msr(E), armA, mph(A)*, *bla*_TEM-1B_, *bla*_OXA-9_, *ant(3’’)-Ia, aac(6’)-Ib, sul2*, *bla*_NDM-5_, *tet(A)*	Col440
KP1679	383	IncFIB–IncHI1B	IncL	*fosA*, *bla*_SHV-145_, *aph(6)-Id, aph(3’’)-Ib, bla*_CTX-M-14b_, *OqxB, OqxA, aph(3’)-VI, qnrS1, mph(E), msr(E), armA, sul2*, *bla*_NDM-5_, *tet(A), mph(A)*, *bla*_OXA-48_	Col440I
KP1601	383	IncFIB–IncHI1B	IncL	*aph(3’)-VI, qnrS1*, *bla*_CTX-M-14b_, *aph(6)-Id, aph(3’’)-Ib, mph(A), aac(6’)-Ib, ant(3’’)-Ia*, *bla*_OXA-9_, *bla*_TEM-1B_, *sul2*, *bla*_NDM-5_, *tet(A), dfrA5, sul1*, *bla*_OXA-48_, *bla*_CTX-M-15_, *mph(E), msr(E)*, *bla*_SHV-145_ *, OqxB, OqxA, fosA*	Col440I
KP1645	383	IncFIB–IncHI1B	IncL	*mph(A), sul2*, *bla*_NDM-5_, *mph(E), msr(E), tet(A), dfrA5, sul1, aac(6’)-Ib, ant(3’’)-Ia*, *bla*_OXA-9_, *bla*_TEM-1B_, *bla*_OXA-48_ *, aph(3’)-Ia, fosA, aph(3’’)-Ib, aph(6)-Id*, *bla*_CTX-M-14b_, *OqxB, OqxA*, *bla*_CTX-M-15_, *qnrS1*, *bla*_SHV-145_	Col440I
KP1663	383	IncFIB–IncHI1B	IncL	*armA, msr(E), mph(E), mph(A), aac(6’)-Ib, ant(3’’)-Ia*, *bla*_OXA-9_, *bla*_TEM-1B_, *sul2*, *bla*_NDM-5_, *tet(A), dfrA5, sul1*, *bla*_SHV-145_, *qnrS1*, *bla*_OXA-48_, *bla*_CTX-M-15_, *aph(3’)-VI, aph(3’’)-Ib, aph(6)-Id, OqxB, OqxA, fosA*, *bla*_CTX-M-14b_	Col440
KP1639	383	IncFIB–IncHI1B	IncL	*mph(A), sul2, mph(E), msr(E), tet(A), sul1, dfrA5, aac(6’)-Ib, ant(3’’)-Ia*, *bla*_OXA-9_, *bla*_TEM-1B_, *bla*_OXA-48_, *bla*_CTX-M-14b_ *, aph(3’)-Ia, OqxA, OqxB*, *bla*_SHV-145_, *aph(3’’)-Ib, aph(6)-Id, qnrS1*, *bla*_CTX-M-15_, *bla*_NDM-5_, *fosA*	Col440
KP1674	383	IncFIB–IncHI1B	IncL	*fosA, tet(A), mph(A), catA1*, *bla*_SHV-145_, *OqxA, OqxB*, *bla*_NDM-5_, *aph(3’)-VI, qnrS1*, *bla*_CTX-M-15_, *aac(6’)-Ib, ant(3’’)-Ia*, *bla*_OXA-9_, *bla*_TEM-1B_, *aph(3’)-Ia, mph(A), sul1, dfrA5, mph(E), msr(E), armA, sul2*, *bla*_OXA-48_, *bla*_CTX-M-14b_, *aph(3’’)-Ib, aph(6)-Id*	Col440I
KP1657	383	IncFIB–IncHI1B	IncL	*fosA, qnrS1, aph(3’)-VI, mph(A), sul2*, *bla*_NDM-5_, *mph(E), msr(E), tet(A), sul1, dfrA5*, *bla*_OXA-9_, *ant(3’’)-Ia, aac(6’)-Ib*, *bla*_OXA-48_, *bla*_TEM-1B_, *bla*_CTX-M-15_, *catA1, aph(6)-Id, aph(3’’)-Ib, OqxA, OqxB*, *bla*_SHV-145_, *bla*_CTX-M-14b_	Col440I
KP1633	383	IncFIB–IncHI1B	IncL	*OqxB, OqxA*, *bla*_SHV-145_, *catA1, mph(A), tet(A), fosA, sul2, armA, msr(E), mph(E), dfrA5, sul1, mph(A), aph(3’)-Ia*, *bla*_TEM-1B_, *bla*_OXA-9_, *ant(3’’)-Ia, aac(6’)-Ib*, *bla*_CTX-M-15_, *qnrS1, aph(3’)-VI*, *bla*_NDM-5_, *aph(6)-Id, aph(3’’)-Ib*, *bla*_CTX-M-14b_, *bla*_OXA-48_	Col440I
KP1710	383	IncFIB–IncHI1B	IncL	*tet(A), dfrA5, sul1, OqxA, OqxB*, *bla*_OXA-48_, *bla*_CTX-M-15_, *aph(3’)-Ia*, *bla*_SHV-145_, *aph(3’’)-Ib, aph(6)-Id, fosA*, *bla*_CTX-M-14b_, *mph(E), msr(E), qnrS1, aph(3’)-VI*, *bla*_TEM-1B_, *bla*_OXA-9_, *ant(3’’)-Ia, aac(6’)-Ib, mph(A), sul2*, *bla*_NDM-5_	Col440I
KP1655	383	IncFIB–IncHI1B	IncL	*fosA*, *bla*_SHV-145_, *aph(3’’)-Ib, aph(6)-Id, OqxB, OqxA*, *bla*_CTX-M-14b_, *bla*_CTX-M-15_, *qnrS1, aph(3’)-VI, tet(A), sul2, mph(E), msr(E), armA, mph(A)*, *bla*_NDM-5_, *sul1, dfrA5, aac(6’)-Ib, ant(3’’)-Ia*, *bla*_OXA-9_, *bla*_OXA-48_	Col440I
KP1608	307	IncFIB–IncFII	–	*fosA6, OqxB, OqxA*, *bla*_SHV-106_, *tet(A*), *bla*_OXA-1_, *aac(6’)-Ib-cr*, *bla*_CTX-M-15_, *dfrA14*, *bla*_TEM-1B_, *aph(6)-Id, aph(3’’)-Ib, sul2, qnrB1, sul1, aadA2, dfrA12, rmtB*, *bla*_NDM-5_	Col440I
KP1734	147	IncFIB–IncHI1B	colKP3	*OqxB, OqxA*, *bla*_SHV-11_, *fosA, aph(3’)-Ia, mph(A), sul1, dfrA5*, *bla*_NDM-1_, *aph(3’)-VI, qnrS1, mph(E), msr(E), armA, sul2, qnrS1*, *bla*_CTX-M-15_, *sul*1, *arr-3, catB3*, *bla*_OXA-1_, *aac(6’)-Ib-cr, qnrS1*, *bla*_CTX-M-15_, *bla*_OXA-232_, *bla*_TEM-1A,_ *bla*_OXA-9_, *ant(3’’)-Ia, aac(6’)-Ib*	Col440I, IncFIB(pKPHS1), IncFIB(pQil), IncR
KP1859	111	IncX3	–	*bla*_NDM-5_, *qnrS1, catA2, OqxB, OqxA, aph(3’)-Ia*, *bla*_SHV-187_, *fosA6, mph(A), sul1, aadA2, dfrA12, aph(6)-Id, aph(3’’)-Ib, sul2*, *bla*_CTX-M-15_	IncFIB(K)
KP1880	101	IncM2	–	*OqxB, OqxA*, *bla*_SHV-106_, *fosA, tet(A), dfrA14, sul2, aph(3’’)-Ib, aph(6)-Id*, *bla*_TEM-1B_, *bla*_CTX-M-15_, *qnrS1*, *bla*_SHV-12_, *bla*_NDM-1_, *sul1, armA, msr(E), mph(E), aac(3)-IId*, *bla*_TEM-1B_	IncFIB(K), IncFII, IncFII(K)
KP1851	39	IncC	–	*bla*_SHV-187_ *, fosA, tet(A), sul1, dfrA7, aph(3’)-Ia, aph(6)-Id, aph(3’’)-Ib, sul2*, *bla*_TEM-1B_, *OqxB, OqxA*, *bla*_CTX-M-15_, *aph(3’)-Ia, sul2, aph(3’’)-Ib, aph(6)-Id, qnrS1*, *bla*_CMY-6_ *, aac(6’)-Ib, sul1, rmtC*, *bla*_NDM-1_	IncFIB(K), IncQ1
KP1917	15	IncC	–	*fosA6*, *bla*_SHV-106_, *bla*_CTX-M-15_, *OqxA, OqxB, aph(3’)-Ia, mph(A), tet(A)*, *bla*_CMY-6_, *aac(6’)-Ib, sul1, rmtC*, *bla*_NDM-1_, *qnrB1, dfrA14*	Col440I, IncFIA(HI1), IncFIB(K), IncFIB(pKPHS1), IncFII(K), IncFII(pMET), IncR

**Table 4:** WGS data representing resistance gene content and plasmid replicons of the *E. coli* isolates.

Sample	Sequence Type	*bla*_NDM_ plasmid	Resistance Genes	Other Replicons
EC1930	648	IncFIB–IncFII	*dfrA12, aadA2, sul1*, *bla*_NDM-5_, *catA1, mph(A)*, *bla*_CTX-M-15_, *tet(B), aac(3)-IIa*, *bla*_OXA-1_, *aac(6’)-Ib-cr*, *bla*_TEM-1B_	Col (BS512), Col (MG828), IncFIA
EC1726	648	IncFIB–IncFII	*mph(A)*, *bla*_NDM-5_, *dfrA12, aadA2, tet(B), dfrA17, aadA5, sul1*	Col (BS512), Col (MG828), Col156, Col440I
EC1609	405	IncFIA–IncFII	*bla*_CMY-42_, *tet(A), dfrA12, aadA2, sul1*, *bla*_NDM-5_, *mph(A)*	p0111
EC1735	405	IncFIB–IncFII	*qepA4, dfrA12, aadA2, sul1*, *bla*_NDM-5_, *mph(A)*, *bla*_TEM-1B_, *tet(B)*	p0111
EC1918	131	IncX3	*bla*_CMY-2_, *bla*_NDM-5_, *bla*_CTX-M-27_, *tet(A), aph(6)-Id, aph(3’’)-Ib, sul2, mph(A), sul1, aadA5*, *bla*_CTX-M-15_	IncFIA, IncFIB(AP001918), IncFII(pRSB107), IncI1
EC1811	2450	IncX3	*bla*_CTX-M-15_, *mph(A), aac(3)-IIa*, *bla*_OXA-1_, *aac(6’)-Ib-cr, aadA5, dfrA17, catA1, tet(B), sul2, aph(3’’)-Ib, aph(6)-Id*, *bla*_NDM-5_	Col (MG828), IncFIA, IncFIB(AP001918)
EC1649	998	IncFIB–IncHI1B	*aph(3’)-VI, qnrS1*, *bla*_CTX-M-15_, *tet(B), sul2, aph(3’’)-Ib, aph(6)-Id*, *bla*_NDM-5_, *sul1, aac(6’)-Ib*, *bla*_TEM-1B_, *bla*_OXA-9_, *dfrA10*	IncFIA, IncFIB(AP001918), IncFII(pRSB107), IncQ1
EC1733	1284	IncX3	*tet(B), sul1, aadA5, dfrA17, mph(A)*, *bla*_CTX-M-15_, *aac(3)-IIa, aac(6’)-Ib-cr*, *bla*_CMY-42_, *bla*_NDM-5_	Col440I, IncFIA, IncFIB(AP001918), IncFII, IncI
EC1825	361	IncFIA–IncFII	*bla*_NDM-5_, *sul*1, *aadA2, dfrA12, catA1, qepA1, mph(A)*, *bla*_OXA-1_, *ant(3’’)-Ia*, *bla*_CMY-145_, *tet(A)*	IncI, IncY
EC1739	167	IncF	*aph(6)-Id, sul2, aph(3’’)-Ib, mph(A)*, *bla*_CTX-M-15_ *, aac(6’)-Ib-cr*, *bla*_OXA-1_, *bla*_CMY-145_, *tet(A), dfrA12, aadA2, sul1*, *bla*_NDM-5_	Col (MG828), IncFIA, IncFIB(AP001918), IncFII, IncI
EC1856	617	IncFII–IncFIA–IncFIB	*bla*_CTX-M-15_, *bla*_OXA-1_, *aac(6’)-Ib-cr, aadA5, sul1*, *bla*_NDM-5_, *bla*_OXA-1_, *aac(6’)-Ib-cr, aadA5, sul1*, *bla*_NDM-5_, *mph(A), sul1, aadA2, dfrA12*, *bla*_OXA-1_, *aac(6’)-Ib-cr, aadA5, sul1, aph(6)-Id, aph(3’’)-Ib, sul2*	Col (BS512), IncFII
EC1507	361	IncFIA–IncFII	*tet(A), bla*_NDM-5_, *sul1, aadA2, dfrA12, mph(A*)	IncI, IncY

Based on the short-read sequencing results, seven *K. pneumoniae* isolates (one isolate each from ST307, ST15, ST147, ST39, and ST101 and two isolates from ST383) and four *E. coli* (one isolate each from ST405, ST131, ST2450, and ST617) were selected for long-read sequencing to have complete circular chromosomes and plasmids for downstream analysis.

**Figure 1:**
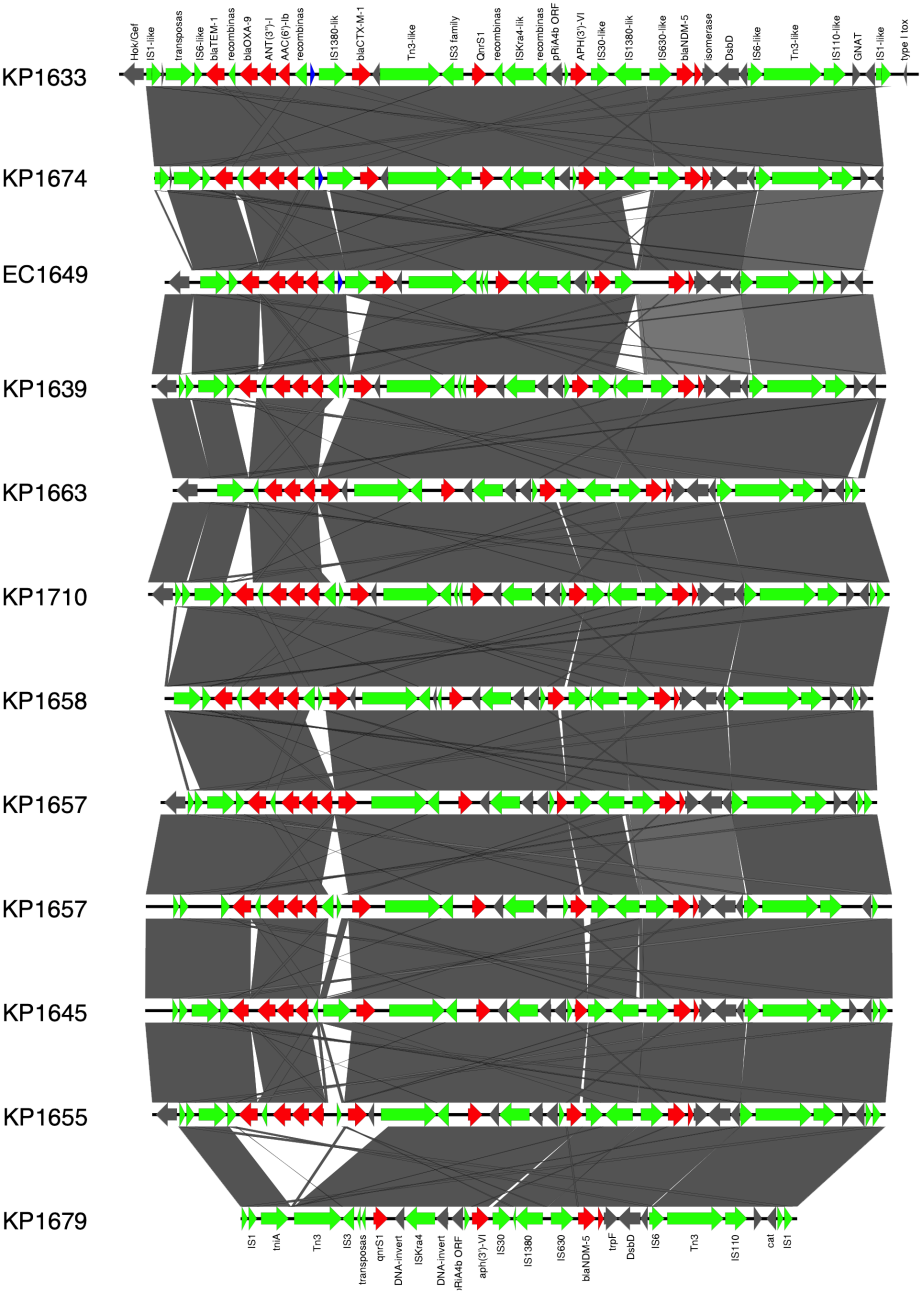
Prophage harboring *bla*_NDM-5_ region comparison, found in ST383 *K. pneumonia* plasmids and in *E. coli* EC1609, with the prophage sequence detected in the long-read sequenced KP1633 isolate. Red represents the resistance genes, green the insertion sequences, transposases in blue DUF domains; and in gray, other genes.

In *K.* pneumoniae, the long-read sequencing of the two ST383, KP1633, and KP1674 showed that the *bla*_NDM-5_ was harbored on a fusion plasmid (IncFIB and IncHI1B) that was 372,845 bp (pKP1633LB_IncFIB_IncHI1B_NDM5) and 372,828 bp (pKP1674LB_IncFIB_IncHI1B_NDM5) in size, respectively. Upon blasting, the plasmid against the NCBI database showed 100% query coverage and 99.9% sequence identity with pFQ61_ST383_NDM_5 that was 376,754 bp in size (accession number CP091814), a plasmid isolated from a clinical *K. pneumoniae* strain in Qatar in 2016. *In silico* plasmid analysis of the rest of the ST383 *K. pneumoniae* isolates that were sequenced using short reads showed that all ST383 isolates carried the same plasmid type. Furthermore, pKP1633LB_IncFIB_IncHI1B_NDM5 carried a 35-kb MDR sequence (from 286,445 bp to 323,218 bp). The MDR region was composed of a transposase from IS*1*, followed by two additional transposases and ends with a transposase. Furthermore, the same MDR region was also detected in isolate KP1674 on the plasmid pKP1674LB_IncFIB_IncHI1B_NDM5 from position 52,157–85,730. In the case of short-read sequenced isolates, the MDR presence was confirmed using blast, and later, a consensus was made for subsequent analysis. Data revealed that this MDR sequence was present in most ST383 isolates, with the exception of isolate KP1679 lacking four resistance genes [*bla*_TEM-1_, *bla*_OXA-9_, *ant(3′)-I*, and *aac(6′)-Ib*]. Similarly, isolate KP1655 noted the absence of *bla*_TEM-1_. In addition to finding missing resistance genes across the isolates, some IS transposases were also found missing (IS*1380-like* only found in three isolates). Interestingly, the starting and ending IS transposases were found in all isolates (**Figure 1**). Furthermore, all ST383 *K. pneumoniae* strains also carried the IncL plasmid harboring *bla*_OXA-48_, which had the same plasmid size (68,942 bp). This plasmid has been reported extensively, and high similarity scores were noticed upon blasting against the NCBI database (Poirel et al., 2012a; Poirel et al., 2012b; Alousi et al., 2018).

Other STs such as ST111 KP1859 carried the *bla*_NDM-5_ on an IncX3 plasmid, while ST307 KP1608 carried the *bla*_NDM-5_ on an IncFII plasmid. On the other hand, the *bla*_NDM-1_ was carried on different Inc plasmids; IncM2 plasmid (pKP1880LB_IncM2_NDM1 87,450 bp) was detected in KP1880 ST 101. This plasmid exhibited 100% query coverage and 100% sequence identity with AR_0127 plasmid (87,450 bp) identified in *a Salmonella enterica* clinical strain in USA in 2018. Moreover, ST39 KP1851 and ST15 KP1917 both carried the *bla*_NDM-1_ on the same IncC plasmid (pKP1851LB_IncC_NDM1 137,593 bp and pKP1917LB_IncC_NDM1 140,300 bp, respectively) (99% query coverage and 99.9% sequence identity). This plasmid exhibited 100% sequence identity and 98% query coverage with pNDM-US (accession number CP006661.1), which was isolated from a clinical strain in 2016 in the USA. Finally, ST147 KP1734 carried the *bla*_NDM-1_ on an IncFIB-IncHI1B plasmid (269,122 bp), while *bla*_OXA-232_ was carried on a ColKP3 plasmid (6,141 bp). The IncFIB–IncHI1B plasmid had similar sequence to those identified in ST383 isolates, KP1633 and KP1674, and the ColKP3 showed the same nucleotide sequence as that of pC06114_4 (accession number CP016038.1) (100% identity and 100% query coverage), a plasmid isolated in 2016 in Germany.

All the characterized *E. coli* isolates carried the *bla*_NDM-5_ gene. Long-read sequencing revealed that ST131 EC1918 and ST2450 EC1811 isolates carried the *bla*_NDM-5_ gene on the same IncX3 plasmid (79,672 bp). Moreover, short-read sequencing showed that ST1284 EC1733 carried an IncX3 plasmid, identical to pKP1859LB_IncX3_NDM5. Moreover, the *bla*_NDM-5_ gene was carried on IncFIA–IncFII (pEC1609LB_IncFIA_IncFII_NDM5, 118,953 bp) in ST405 EC1609 and on IncFII–IncFIA–IncFIB (pEC1856LB_IncFII_IncFIA_IncFIB_NDM5, 118 140,386 bp) in ST617 EC1856. Short-read sequences scaffolding showed that IncF plasmids (with different structures and sequences) carrying *bla*_NDM-5_ gene was found in the rest of *E. coli* despite STs.

ST383 *K. pneumoniae* virulence factor analysis revealed the presence of virulence genes responsible for acriflavine resistance, type VI secretion system, synthases, transporters, esterase, adherence, regulation, immune modulation, fimbriae, and delivery. Some genes were found to be missing, for example, the regulatory gene for the mucoid phenotype *rmp*A2 (KP1710, KP1679, KP1663, KP1657, KP1658, KP1655, KP1645, KP1639, and KP1601). In *E. coli*, the presence of the virulence genes was associated with acriflavine resistance, invasion, secretion, adherence, fimbriae, enterobactin, and yersiniabactin. Notably in ST648 EC1930 in comparison with ST648 EC1726, some virulence factors were found missing: *fim* genes for fimbriae, *gsp* genes for secretion, *irp* genes for capsule and invasion characteristics, and *ybt* genes for yersiniabactin. In addition, in comparison between the ST405 EC735 and ST405 EC1609, genes of *esp* variations responsible for secretion and two genes for adherence (*pap*E and *pap*F) were found to be missing (**Supplementary Tables S1**, **S2**).

### Phylogeny and clonality

**Figure 2:**
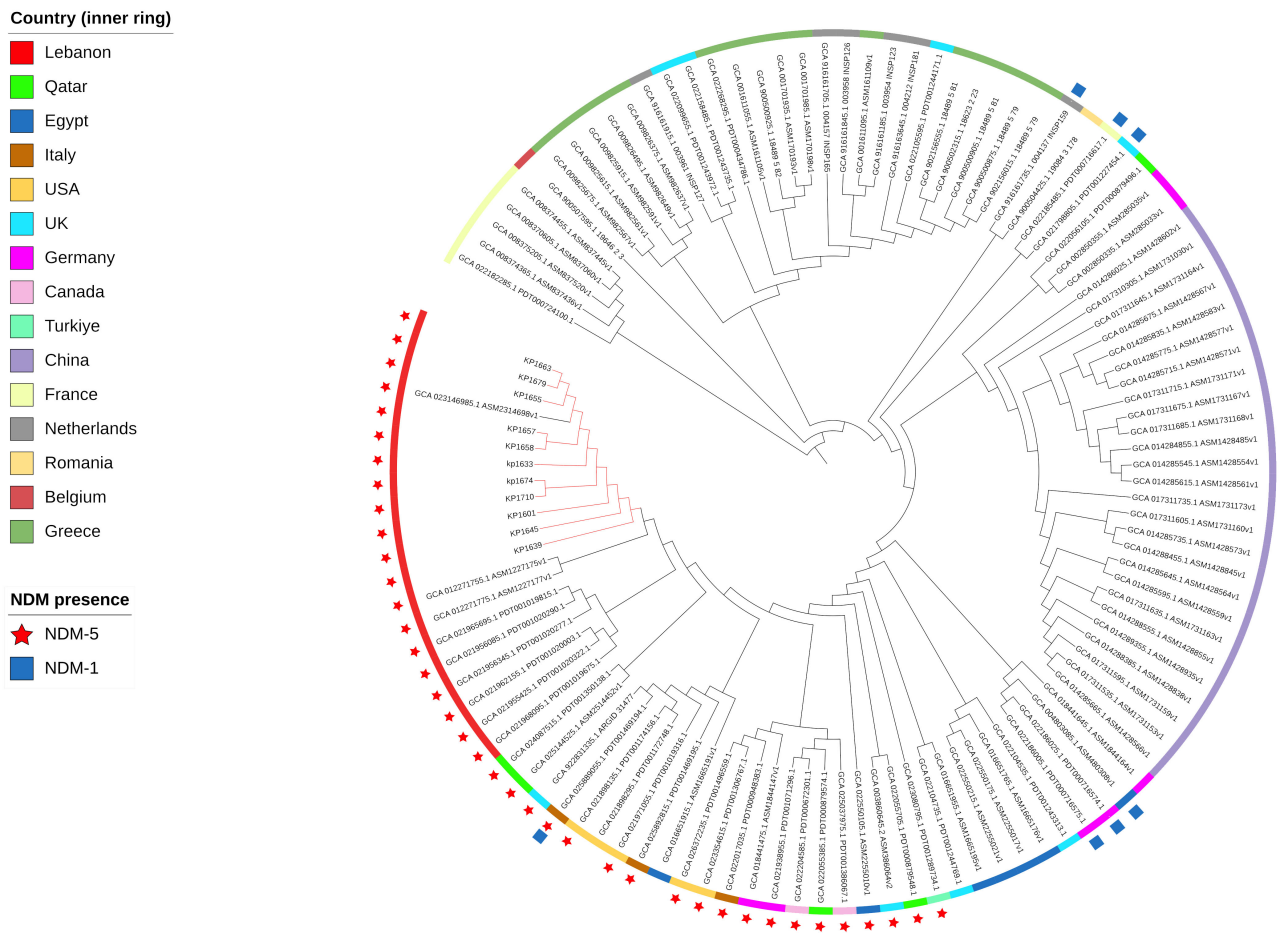
SNP-based phylogeny for global *K. pneumoniae* ST383 with 105 isolates downloaded from NCBI database. Red nodes highlight the isolates used in this study.

To evaluate the relatedness of the *K. pneumoniae* ST383 isolates in the study, global SNPs phylogeny was performed on all *K. pneumoniae* ST383 isolates in the NCBI database. The phylogenetic tree showed that our ST383 isolates clustered together along with previously detected NDM-5 producing ST383 isolates from Lebanon (**Figure 2**). Furthermore, SNP analysis was performed on all isolates belonging to this clade and showed high similarity with SNPs ranging from 11 to 73 (read, **Table 5** and **Supplementary Table S3**). Plasmid analysis of the Lebanese clade showed that all the clustered isolates carried the *bla*_NDM-5_ gene on the same Inc plasmid (IncFIB–IncHI1B) previously detected. Remarkably, these clustered isolates were all NDM-5/OXA-48 co-producers, and their *bla*_OXA-48_ gene was similarly carried by an IncL plasmid (**Figure 2**).

**Table 5:** Number of SNPs in the *K. pneumoniae* ST383 isolates in this study as compared to the reference KP1674.

Isolate	Number of SNPs
KP1633	15
KP1679	26
KP1655	28
ASM2314698v1	31
KP1601	41
KP1710	50
ASM1227177v1	55
KP1658	55
ASM1227175v1	61
KP1663	61
PDT001019815.1	71
PDT001020290.1	71
PDT001020277.1	73
PDT001019675.1	78
PDT001020322.1	80
PDT001020003.1	83
KP1657	90
KP1645	91
KP1639	100

**Figure 3:**
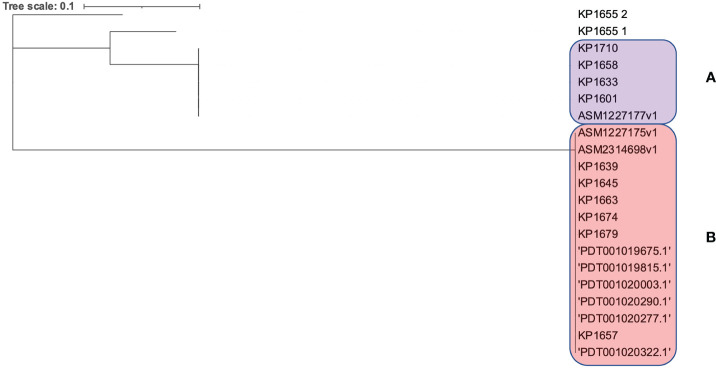
SNPs based phylogeny for the CRISPR/Cas arrays of the Lebanese ST383 *K. pneumoniae* isolates. **(A)** represents the clade in the pink while **(B)** represents the clade in purple.

Moreover, the clonality of the *K. pneumoniae* ST383 strains was assessed through analyzing the CRISPR array sequences. A population of any isolates that went through the same environmental conditions (subjected to phages or plasmids) will have a similar CRISPR array, since these sequences (correlated with foreign sequences) are added in chronological order (Gagaletsios et al., 2022). CRISPRCasFinder showed that all genomes have the CRISPR/Cas I-E type. The sequences were then aligned, and a SNPs-based phylogenetic tree was executed (**Figure 3**). The tree showed that the isolates were divided between two distinct clades (A and B) and one isolate (KP1655) that has two CRISPR arrays residing alone. The two clades were composed of both isolates from this study and previously identified in Lebanon. This result shows that the Lebanese isolates’ population is originating from at least three different ancestors, which underwent different environmental conditions.

## Discussion

Lebanon confronts significant antibiotic resistance, notably with the recent emergence of resistance to ceftazidime-avibactam in CRE treatments (Araj et al., 2018; Iskandar et al., 2021; Rima et al., 2022; Zakhour et al., 2024),. This study investigated CR *K. pneumoniae* and *E. coli* strains exhibiting resistance to last line antibiotic choices, including carbapenems and CZA, as reported by (Sobh et al., 2021). It intended to explore mechanisms involved in their resistance and dissemination especially through characterizing the genetic features of *bla*_OXA-48-like_ and/or *bla*_NDM_-positive *K. pneumoniae* and *E. coli* isolates recovered at a major Lebanese medical center. Sequence data showed the presence of *bla*_NDM_ on plasmids of different Inc groups, in *K. pneumoniae* belonging to different STs (**Table 3**). Most of the *K. pneumoniae* isolates belonged to ST383 and carried a multireplicon plasmid (IncFIB–IncHI1B) with *bla*_NDM-5_. This sequence type has been reported in different parts of the world, including countries around the Mediterranean basin, where it contributes to the burden of CRE (Turton et al., 2018; Edward et al., 2022; Tsui et al., 2023). The detection of *bla*_NDM-5_ positive isolates raises significant concerns due to its potent ability to break down a broad spectrum of β-lactam antibiotics, including carbapenems. Recognized globally for its contribution to antibiotic resistance (Daaboul et al., 2023; Linkevicius et al., 2023; Simner et al., 2023; Tao et al., 2023), the prevalence of *bla*_NDM-5_ underscores the urgent challenge it poses to treating bacterial infections. The concurrent presence of *bla*_NDM-5_ and *bla*_OXA-48_ in our strains severely limits treatment options with last-resort antibiotics like ceftazidime-avibactam. This synergy of resistance genes, documented across the MENA region (Dandachi et al., 2016; Aqel et al., 2017; Rojas et al., 2017; Solgi et al., 2020; Ahmed El-Domany et al., 2021) and mirrored in findings from Italy (Lorenzin et al., 2022), emphasizes the global nature of this threat and underscores the need for international efforts in surveillance and containment. This finding highlights the important role of horizontal transfer in the worldwide spread of *bla*_NDM_ resistance determinants (Paskova et al., 2018).

Additionally, the close clonal phylogenetic relationship of ST383 isolates was confirmed by SNPs phylogeny. ST383 is a high-risk clone associated with the spread of diverse resistance mechanisms (Papagiannitsis et al., 2010) like *bla*_KPC_ and *bla*_VIM_. Nevertheless, CRISPR-Cas array analysis revealed genetic diversity within ST383 *K*. *pneumoniae* isolates, identifying distinct clades and indicating diverse ancestral origins, suggesting varied exposure to environmental pressures and selective forces.

On the other hand, *bla*_NDM-5_ was also found in *E. coli* isolates. *E. coli* isolates, which belonged to diverse STs, harbored a variety of *bla*_NDM-5_-carrying plasmids. The variation in plasmids involved in the spread of *bla*_NDM-5_ underlines the important role of mobile elements, like insertion sequences and transposons, in the horizontal spread of resistance mechanisms. However, short-read sequencing data suggested that, in most of the *E. coli*, which belonged to diverse STs, the *bla*_NDM-5_ gene was localized on IncF-type plasmids. Additionally, WGS data confirmed the presence of similar *bla*_NDM-5_-carrying plasmids, like pKP1674LB_IncFIB_IncHI1B_NDM5 (IncFIB and IncHI1B) and pEC1609LB_IncFIA_IncFII_NDM5 (IncF-type), into different STs or species. These findings highlight the important role of plasmids in the horizontal gene transfer (HGT) of resistance genes in the institution and community where such strains exist.

## Conclusion

In conclusion, the current study confirmed the fact that production of NDM carbapenemases is one of the most clinically significant resistance mechanisms among CRE isolates in Lebanon, including ceftazidime-avibactam. This finding is in line with previous studies highlighting the importance of *bla*_NDM_ spread, canceling last-line therapeutic options worldwide (Chudejova et al., 2021). Additionally, our study showed the contribution of divers STs and mobile genetic elements to the success of NDM resistance mechanism. Furthermore, the high-risk clone ST383 and the broad host range plasmids IncH and IncF has contributed to the successful prevalence and dissemination of NDM resistance. Thus, such findings emphasize the need of scrutinizing the implementation of infection control aspects to curb/control the spread of these “superbugs”.

## Data availability statement

The datasets presented in this study can be found in online repositories. The names of the repository/repositories and accession number(s) can be found in the article/**Supplementary Material**.

## Ethics statement

Ethical approval was not required for the study involving humans in accordance with the local legislation and institutional requirements. Written informed consent to participate in this study was not required from the participants or the participants’ legal guardians/next of kin in accordance with the national legislation and the institutional requirements.

## Author contributions

GS: Methodology, Writing – review & editing. GA: Supervision, Writing – review & editing. MF: Data curation, Formal analysis, Software, Visualization, Writing – original draft. TS: Formal analysis, Investigation, Methodology, Writing – original draft. JH: Funding acquisition, Resources, Writing – review & editing. CP: Conceptualization, Data curation, Formal analysis, Investigation, Validation, Writing – original draft, Writing – review & editing. ME: Writing – review & editing. IB: Conceptualization, Formal analysis, Funding acquisition, Investigation, Project administration, Supervision, Visualization, Writing – original draft, Writing – review & editing.
