# Corrigendum: Molecular characterization of carbapenem and ceftazidime-avibactam-resistant Enterobacterales and horizontal spread of *bla*_NDM-5_ gene at a Lebanese medical center

**DOI:** 10.3389/fcimb.2024.1466718

**Published:** 2024-07-31

**Authors:** Ghena Sobh, George F. Araj, Marc Finianos, Tsolaire Sourenian, Jaroslav Hrabak, Costas C. Papagiannitsis, Mira El Chaar, Ibrahim Bitar

**Affiliations:** ^1^ Department of Pathology and Laboratory Medicine, American University of Beirut Medical Center, Beirut, Lebanon; ^2^ Department of Microbiology, Faculty of Medicine, University Hospital in Pilsen, Charles University, Pilsen, Czechia; ^3^ Department of Microbiology, University Hospital of Larissa, Larissa, Greece; ^4^ Faculty of Health Sciences, University of Balamand, Beirut, Lebanon

**Keywords:** *Escherichia coli*, *Klebsiella pneumoniae*, ST383, *bla*_NDM-5_, carbapenem resistance

In the published article, an author name was incorrectly written as ‘Costas C. Pappagianitsis’. The correct spelling is ‘Costas C. Papagiannitsis’.

The authors apologize for this error and state that this does not change the scientific conclusions of the article in any way. The original article has been updated.

